# Effect of monoethanolamine salt-containing dicarboxylic acid and plant growth regulators on the absorption and accumulation of mercury

**DOI:** 10.1016/j.sjbs.2022.02.035

**Published:** 2022-02-25

**Authors:** Anna Makarova, Elena Nikulina, Nina Tsirulnikova, Ksenia Pishchaeva, Andrey Fedoseev

**Affiliations:** a D. Mendeleev University of Chemical Technology of Russia, Miusskaya Sq., 9, 125047 Moscow, Russia; b Institute of Chemical Reagents and Special Purity Chemicals of the National Research Center Kurchatov Institute (IREPC), St. Bogorodsky Val, 3, 107076 Moscow, Russia

**Keywords:** Phytoextraction of mercury, Complexones, Heavy metals, Dicarboxylic acid

## Abstract

In the modern world, mercury has become an extremely dangerous pollutant due to intensive human activity. Currently, sources of mercury are wastes from chemical industries, as well as mines, oil combustion products, and household waste. Phytoextraction of heavy metals from soil is considered one of the most promising and cost-effective technologies. The efficiency of this process can be increased by introducing various amendments. The use of additives in phytoextraction can enhance the absorption of heavy metals and increase their concentration in various parts of the plant. This article presents the results of a study of various chelating agents for effective phytoextraction of mercury with white clover (*Trifolium repens L.*) and watercress (*Lepidium sativum*). In the present study, the monoethanolamine salt of dithiodiacetic acid (MEDBA) was used. The optimal concentration of MEDBA on watercress and creeping clover has been determined for highly efficient phytoextraction of mercury. Research has been carried out with a complex of exogenous growth regulators (GA / IAA / Fe-EDDHA). The results showed that the use of phytohormones and plant growth regulators led to a synergistic effect in combination with thiosulfate, but a pronounced inhibitory effect was observed with the use of MEDBA.

## 1 Introduction

Mercury is an extremely dangerous pollutant. As a result of intense human activity, it can be found in high levels in the environment (Wang et al., 2012, Gaur et al., 2014). Mercury has high geochemical mobility owing to its volatility, atomic form, sublimation at moderate temperatures, and solubility of its sulfides in alkaline hydrothermal waters (Azevedo and Rodriguez, 2012, Sobral-Souza et al., 2019).

Currently, mercury is widely used in metallurgical, chemical, electrical, pulp, and paper industries (ACAP, 2005; Maxon, 2005), as well as in medicine and pharmaceuticals. Waste and emissions from these industries are among the most toxic environmental pollutants (UNEP, 2018). Other sources of mercury pollution are mercury mines, combustion of oil and coal, municipal waste, and fungicides (Mukherjee et al., 2004, Driscoll et al., 2013).

Considered to be the safest and most economical approach to reduce mercury levels, the phytoremediation of heavy metal-contaminated soils has been extensively studied (Hazrat et al., 2013, Liu et al., 2018a, Liu et al., 2018b). Considerable research has been devoted to the phytoextraction of heavy metals and approaches that increase the process efficiency (DalCorso et al., 2019). From this perspective, mercury phytoextraction has been comprehensively explored (Lomonte et al., 2011, Franchi et al., 2017), and studies have investigated the influence of chemical compounds and other additional reinforcing components on mercury accumulation in plant tissues (Wang et al., 2012).

Several authors studied the inducing effect of sulfur-containing inorganic compounds such as sodium and ammonium thiosulfates during mercury phytoextraction (Moreno et al., 2005). These chelators are effective because of the high chemical affinity of mercury for the sulfur atom and the formation of selective complex soluble compounds ([Me(S_2_O_3_)]^2-^).

The influence of carboxyl containing complexones (e.g. ethylenediaminetetraacetic acid [EDTA], diethylenetriaminopentaacetic acid [DTPA], nitrilotriacetic acid [NTA], on mercury accumulation has also been tested. Notably, polyaminopolycarboxylic acids are versatile in complexation with most metal cations and are quite effective in polymetallic contamination of native soils (Qian et al., 2018)

Additionally, several studies have explored the combination of amplifying components in a general assistance scheme with inclusion in complex processing, such as plant growth-promoting bacteria (PGPR) and exogenous phytohormones (cytokinin) (Cassina et al., 2012, Franchi et al., 2017).

A recent publication of the proposed study described a new chelating agent that represents the class of polycarboxylic acids. The monoethanolamine salt of bithiodiacetic acid, in which the sulfur atom acts as a coordination partner for mercury, creates a high selectivity of interaction (Makarova et al., 2021a). According to data obtained using meadow clover (*Trifolium repens* L.), the coefficient of biological accumulation (the concentration of the test substance in/on the test organism referred to the concentration of the test substance in the environment) with the addition of monoethanolamine salt of 2,2′-(ethylenedithio) diacetic acid (MEDBA) was higher than that of EDTA and sodium thiosulfate. This effect was verified despite the significant plant stress caused by the high concentration of the reagent. Furthermore, the same study reported significant positive effects using additional growth-regulating components: auxin (4 (indole-3-yl) butyric acid [IAA], gibberellic acid [GA], and iron chelate [sodium salt of ethylenediamine N, Nʹ-bis(hydroxyphenyl) acetic acid of iron Na(Fe-EDDHA)]). These components were used in combination with a phosphorus-containing chelating agent, the potassium salt of 1-hydroxy ethylidene-1,1-diphosphonic (K_2_HEDP). Additional treatment with a complex of growth-regulating substances increased the plant biomass (up to 27%) and mercury absorption (up to 75%).

This study investigated the effectiveness of MEDBA in the phytoextraction of mercury. We used *T. repens* as a culture to determine the optimal concentration of a complexone for introducing into the soil substrate. We also studied the effect of additional growth-regulating substances (auxins, gibberellins, and iron chelate) combined with chemical amendments (such as MEDBA) to compare the effectiveness of the complex scheme in combination with the most common inducer, thiosulfate. The experiments were conducted using *L. sativum* as described in previous studies (Smolinska et al., 2006, de Matos et al., 2021).

## 2 Materials and methods

### 2.1 1. Material and chemicals

Samples of MEDBA aqueous solutions (with a mass content of the target component of 20% and iron chelate with a mass content of the target component of 4%) were provided by the Laboratory for the Technology of Complexones and Complex Compounds of the Research Center ‘Kurchatov Institute’—IREA (Institute of Chemical Reagents and Highly Pure Chemical Substances). Analytical analyses of the reagent samples was performed using equipment of NRC ‘Kurchatov Institute’—IREA Shared Knowledge Center. Growth regulators (PGRs) IAA and GA (5.5 g/kg) were acquired from SELHOZEKOSERVICE LLC (Russian Federation, commercial name ‘Zavyaz’) and Orton LLC (Russian Federation, commercial name ‘Kornevin’), respectively. Sodium thiosulfate was purchased from RusChem (Russian Federation).

### 2.2 Research plants

*T. repens* was used in experiments in the first stage (Makarova et al., 2021a). The positive qualities of *T. repens* in this study are winter resilience hardiness for the Russian climate and ability to produce sufficient biomass. Second-stage experiments were performed with *Lepidium sativum*, which is also notable for its unpretentiousness, low nutrient requirements, and short vegetative cycle (Smolinska et al., 2010, Smolinska and Rowe, 2015).

### 2.3 Soil preparation

For the vegetation study, pots measuring 11.5 × 10.8 cm were filled with three-fourth parts of soil. The contents of the pots were then divided into two parts and poured into two separate containers.

An aqueous solution was used to simulate mercury contamination by Hg(NO_3_)_2_·H_2_O. For that, Hg(NO_3_)_2_·H_2_O (9.86 ± 0.14 mg) was used for a vegetation experiment with an excess of the maximum permissible concentration (MPC) of mercury in the soil by a factor of 5. A weighed portion of Hg(NO_3_)_2_·H_2_O was transferred to a burette, 10 ml of distilled water was added, and the solution was added to one of the containers. For a vegetation experiment with ten times excess of the MPC level of mercury in the soil, 10 ml of distilled water was added to Hg(NO_3_)_2_·H_2_O (19.74 ± 0.26 mg), and the solution was added to the other container. Each burette was rinsed with distilled water, and then the solution was added to the corresponding container with soil.

To remove the excessive moisture, the soil containing mercury nitrate was poured onto filter paper. In the first stage of the study, the soil was dried for 1 week, and in the second stage, the soil was dried for 4 weeks under natural conditions, at a temperature of 22 ± 2 °C.

Pots were filled with the dried soil.

Nitrogen–phosphorus–potassium fertiliser (NPK) concentration in the pot was 0.3 mg/ml soil.

### 2.4 Potted experiment to determine the effects of soil amendments

Model experiments were performed as described in a previous study (Makarova et al., 2021).

A pot with universal soil and fertiliser without mercury was used as a control. Universal soil contains peat, NPK, limestone components, rippers, and organic-matter content of at least 70%. In each pot, 20 seeds of *T. repens* were planted in the first stage, and 50 seeds of *L. sativum* were planted in the second stage. After sowing, the seeds were sprinkled with 5–10 g of soil and watered with approximately 50 ml of tap water. On days 5–7, seed shoots were observed, and morphometric and physical parameters (plant growth and number of shoots) of the growing seeds were measured; these data were recorded and entered into an Excel file.

The optimal concentration of MEDBA was determined at three values: 1, 5, and 10 mmol/kg DW’ MEDBA, which was added to the soil substrate. Further studies on the effect of combined induction were performed according to the scheme presented in Table 1. To reduce the load on the seedlings, chemical inductors were introduced into the substrate using the split method; the calculated dose was divided into five equal parts, applied sequentially for 5 days starting from the 26th day after planting.

**Table 1:** Scheme for the use of chemical inductors for phytoextraction of mercury from a contaminated substrate.

**Reagent**	**Concentration**	**Treatment type**	**1st day of processing**	**Number of treatments**	**Interval between treatments, number of days**
MEBTA	0.1 ml/l	under the root, split	26	5	1
Na_2_S_2_O_3_	0.132 mg/l	under the root, split	26	5	1
GA (‘Zavyaz’)	100 mg/l	by shoots	12	3	8
IAA (‘Kornevin’)	200 mg/l	by shoots	12	3	8
Na(FeEDDHA)	1 ml/l	by shoots	12	3	8

All experiments were prepared using triplicates. The plants were removed 33 days after sowing. The removed *T. repens* seedlings were cleared of soil and washed with water; their shoots and roots were separated, dried for three days at 22 ± 2 °C, and weighted.

### 2.5 Analysis of mercury content in plant parts

The mercury content of the samples was determined using inductively coupled plasma–mass spectroscopy (ICP–MS). (ICP-MS; X-7, Thermo Elemental, USA). The following operating parameters were used: generator output power, 1250 W; concentric nebuliser PolyCon; spray chamber in quartz cooled until 3 °C; flow rate of the plasma-forming flow, Ar 13 l/min; auxiliary flow, Ar 0.9 l/min; Ar flow rate in the atomiser, 0.89 l/min; flow rate of analysed samples, 0.8 ml/min; and resolution, 0.8 M. The relative standard deviation for all elements did not exceed 0.3 when measuring the content of these elements up to 5 detection limit (DL) and did not exceed 0.15 when measuring the content > 5 × DL.

The remaining soil was dried, and its mass and mercury content were measured. To determine the mercury content in the dried soil samples, portions of the analysed samples weighing 200 mg were placed in Teflon cups, wetted with a mixture of hydrochloric (36%) and nitric acids (70%), and boiled for 5 min. Subsequently, 5–10 cm^3^ of water was added. The resulting solutions were transferred into polyethylene bottles and diluted with water.

### 2.6 Calculation of bioconcentration (BAF) and translocation (TF) factors

The coefficients of BAF and TF were estimated according to the methods described in previous studies (Cojocaru et al., 2016, Makarova et al., 2021a).

### 2.7 Statistical analysis

All determinations were performed in triplicates, and the results of the chemical and plant tests were obtained in Microsoft Excel (Microsoft, USA). The table reveals the arithmetic mean values and standard errors of the mean inherent in the values of the estimates according to the Student’s criterion.

## 3 Results

### 3.1 Determination of the optimal concentration of the inducing agent MEDBA

**Fig. 1:**
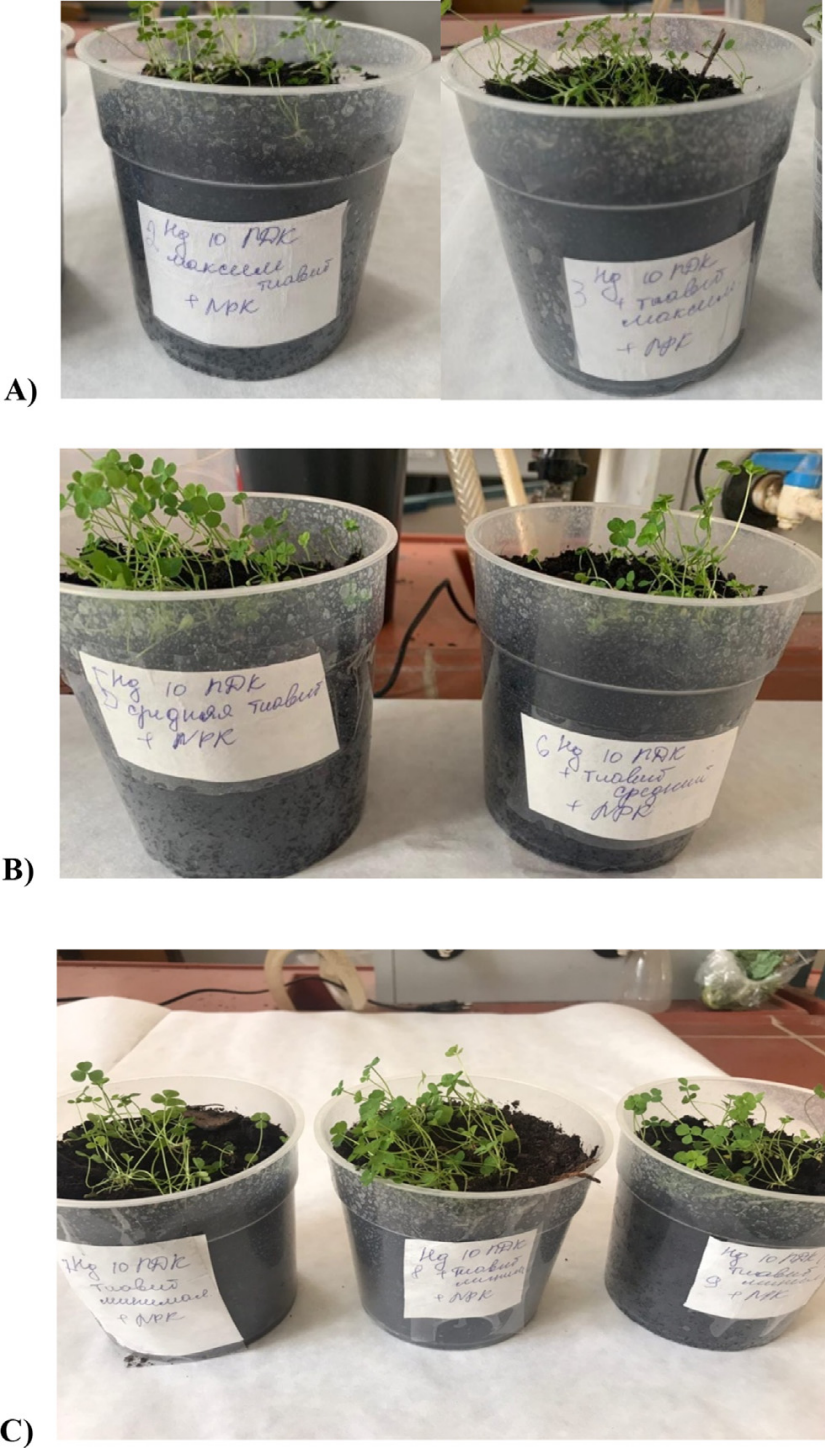
Photographs of *T. repens* at different amounts of MEDBA introduced into the substrate with a mercury contamination level of 10 MPC: A) maximum; B) average; C) low.

The results showed that the conditions of the seedlings were satisfactory at all three concentrations of MEDBA. Fig. 1 shows *T. repens* grown under different concentrations of MEDBA in the substrate containing a mercury contamination level of 10 MPC.

At the maximum MEDBA concentration, the lowest plant biomass was observed at both 5 MPC and 10 MPC contamination levels. Additionally, some leaves exhibited slight signs of chlorosisand wilting. The highest biomass accumulation was shown by the treatment in which the correction was made at 0.1 mM.

Table 2 presents the overall growth rates and mercury content in various plant parts. Compared to the control, the plant biomass under 5-MPC mercury contamination level and MEDBA treatments at average and low doses increased by 65% and 40%, respectively. At a mercury contamination level of 10 MPC with average and low concentrations of MEDBA, plant biomass increased insignificantly. However, at the maximum dose of MEDBA, plant biomass decreased by approximately two times (44%). Despite a significant increase in mercury concentration in the shoots (218%) at this level of pollution, high doses of MEDBA resulted in an insignificant (11%) increase in the absorption of the pollutant.

**Table 2:** Results of phytoextraction of mercury by *T. repens* when applying different concentrations of MEDBA.

**Concentrations of MEDBA, mM**	**Hg**	**Plant weight, g**		**Hg concentration, μg / g**		**Total amount Hg, μg / g**		
		**Shoots**	**Roots**	**Shoots**	**Roots**	**Shoots**	**Roots**	**Entire plants**
	
maximum	10 MPC	0.036 ± 0.03	0,007 ± 0,001	137.4 ± 0.002	719 ± 0.002	4.95 ± 0.002	5.03 ± 0.002	9.98 ± 0.002
	5 MPC	0.042 ± 0.014	0.007 ± 0.001	39.9 ± 0.002	538 ± 0.002	1.68 ± 0.002	3.77 ± 0.002	5.44 ± 0.002
average	10 MPC	0.069 ± 0.027	0.011 ± 0.004	97.7 ± 0.002	710 ± 0.002	6.74 ± 0.002	7.57 ± 0.002	14.31 ± 0.002
	5 MPC	0.082 ± 0.025	0.012 ± 0.002	35.9 ± 0.002	344 ± 0.002	2.96 ± 0.002	4.13 ± 0.002	7.08 ± 0.002
low	10 MPC	0.067 ± 0.024	0.011 ± 0.002	115 ± 0.002	719 ± 0.002	7.67 ± 0.002	7.67 ± 0.002	15.34 ± 0.002
	5 MPC	0.072 ± 0.021	0.008 ± 0.001	28.6 ± 0.002	336 ± 0.002	2.06 ± 0.002	2.8 ± 0.002	4.86 ± 0.002
control	10 MPC	0.067 ± 0.01	0.010 ± 0.005	63 ± 0.002	468 ± 0.002	4.20 ± 0.002	4.84 ± 0.002	9.04 ± 0.002
	5 MPC	0.048 ± 0.008	0.009 ± 0.006	55.2 ± 0.002	343 ± 0.002	2.65 ± 0.002	2.97 ± 0.002	5.62 ± 0.002

**Fig. 2:**
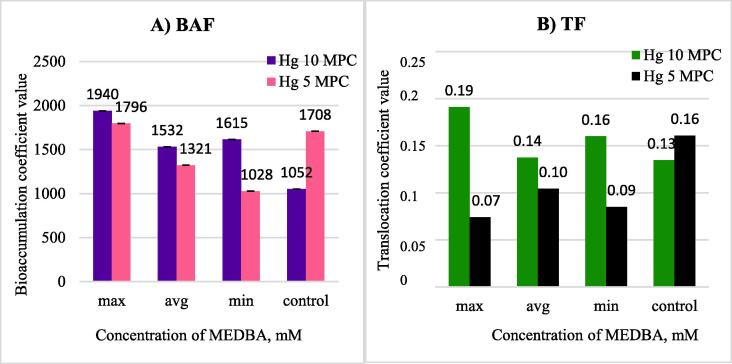
Bioaccumulation (A) and translocation (B) coefficients at various MEDBA concentrations.

Notably, the greatest decrease in biomass occurred in the belowground parts of plants: 14% and 46% with mercury contamination levels of 5 MPC and 10 MPC, respectively. According to the data obtained, there were some differences in mercury absorption at different levels of contamination. At a relatively low mercury contamination level (5 MPC), the introduction of the chelating agent MEDBA was not associated with a high phytoaccumulation. In contrast, mercury concentration in plant parts was 37% lower than that of the control; however, increased biomass ultimately compensated for the total mercury accumulation. At the highest mercury contamination level (10 MPC). MEDBA was effective at all concentrations. Average and low doses of MEDBA application ensured the development of biomass and increased mercury absorption in belowground parts by 55% and 82.5%, respectively, and the total amount of phytoextracted mercury by 58.3% and 69.6%, respectively. Fig. 2 presents data on the obtained BAF and TF at various concentrations of the chemical additive.

The addition of MEDBA at any concentration increased mercury absorption rates, particularly for the maximum applied dose of mM, at which the bioaccumulation coefficient increased by 1.84 times compared to phytoextraction without chemical induction. Moreover, the highest translocation coefficient value, 0.19, was achieved at the maximum concentration MEDBA; this exceeded the translocation coefficient value of the control by 1.46 times. At the average and low MEDBA concentration, less pronounced effects were observed; however, the smallest increase in both coefficients was shown by the introduction of MEDBA at an average concentration. Notably, the effect of MEDBA as an inducer of phytoextraction did not decrease with the increasing concentration; however, the specific absorption of mercury increased. With an increase in the dose of the reagent, there was clear inhibition of plant growth, which ultimately affected the total amount of mercury removed, thereby reducing the efficiency of the process as a whole. We assume that this behavior is directly related to the chemical formula of the inductor, which contains monoethanolamine known for its biological activity (C_2_H_7_NO) (Fujii et al., 1972, Isaev, 1981). In this context, prior studies have revealed that ethanolamines, particularly monoethanolamine, play an important role in redox reactions and that the plant growth regulators enhance the activity of the enzymatic systems and protect plants against stress (Ilker et al., 1976, Horvath et al., 1985, Eckert et al., 1988).

As other growth regulators, MEDBA is characterised by threshold values of the effective concentrations, above which the opposite effect, inhibition, is manifested. Thus, under mercury contamination of approximately 10 MPC, the maximum application dose cannot be recommended for practical use despite the high rates of bioaccumulation and translocation; thus, it is better to adhere to averige–low concentrations. At 5-MPC contamination, the best overall mercury removal was achieved with the average dose of the chelating agent.

### 3.2 Combination of chemical and PGR for mercury phytoextraction

#### 3.2.1 Effect of adjustments on plant growth and biomass

This study on the treatment combinations of the chemical reagents and PGRs was performed at an average dose of MEDBA and contamination of the soil substrate with mercury at 5 MPC. Table 3 presents the measured biometric parameters of the plants in all treatments.

**Table 3:** Plant growth and biomass as a result of obtained by applying all treatments.

**Induction type**	**Average number of seedlings in a pot. pcs.**	**Average seedling height. cm**	**Average number of leaves per plant. pcs**	**Dry biomass. g**		
				**Shoots**	**Roots**	**Plants**
Control (NPK)	27	7.5	3.5	0.173	0.054	0.227
MEDBA/ NPK	34	8.2	3.3	0.236	0.109	0.345
MEDBA/ GA/ IAA/ Fe- chelate/ NPK	28	7.7	3.4	0.188	0.123	0.311
Na_2_S_2_O_3_/ NPK	24	7.6	3.6	0.163	0.096	0.259
Na_2_S_2_O_3_/ GA/ IAA/ Fe- chelate/ NPK	30	7	3.4	0.211	0.087	0.298

All applied induction complexes exhibited a stimulating effect on the growth of biomass of watercress sprouts, from 14% to 52%. The greatest increase in biomass compared to the control was observed in plants treated only with the monoethanolamine salt of dithiobioacetic acid (52%) and the smallest (14.1%) with the use of thiosulfate. The supplementation of MEDBA with GA, IAA, and iron chelate resulted in decreased plant biometric parameters compared to pure MEDBA treatment: sprout height and biomass of the belowground part and of the whole plant (6%, 20%, and 10%, respectively). The exception was root biomass, which was 12.8% higher than that of the control. However, an overall increase in biomass was observed using the combined treatment with PGRs (MEDBA/GA/ IAA/Fe-chelate/NPK), which was 37% higher than that of the control. The opposite effect was observed when thiosulfate was used for chemical correction. The combination of thiosulfate with PGRs (GA/IAA/Fe-chelate) led to increased biometric parameters, both in comparison with the control and with only thiosulfate treatments, particularly in relation to belowground parts. For example, the shoot mass was 29% and 22% higher than that of thiosulfate and control, respectively. Supplementing thiosulfate with the PGR complex made it possible to increase the biomass by up to 31% compared with the control.

#### 3.2.2 Effect of corrections on mercury absorption and accumulation

**Fig. 3:**
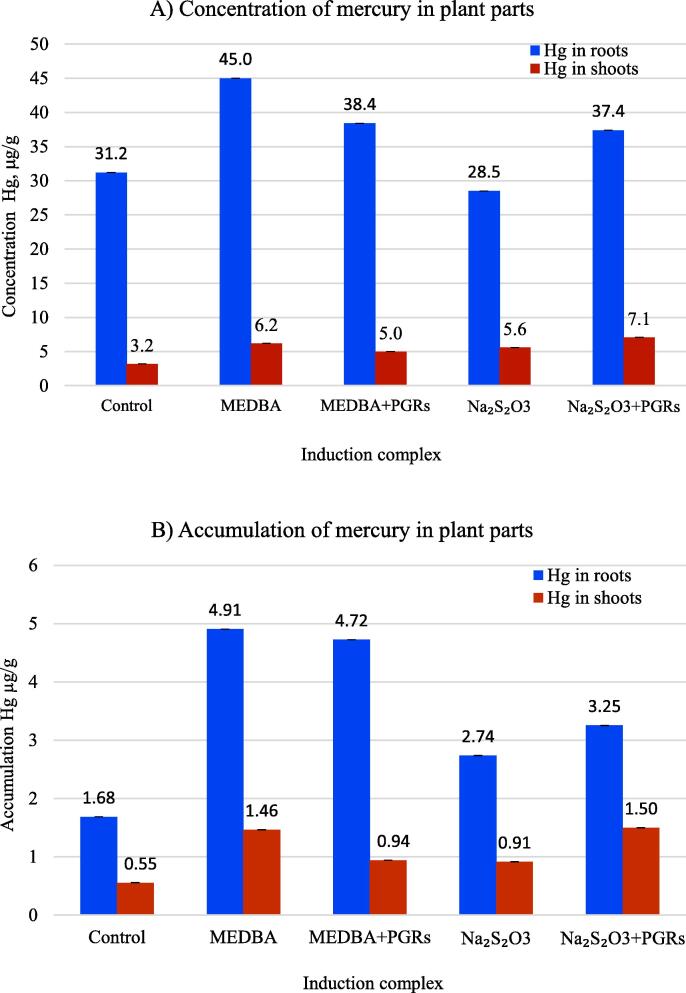
Characteristics of absorption and accumulation of mercury in plant parts using various induction complexes: A) concentration of mercury in plant parts; B) accumulation of mercury in plant parts.

An effect similar to that of adjustments on plant growth and development was recorded for mercury absorption. Any of the tested complexes of additives contributed to an increase in mercury absorption as well as phytoaccumulation (Fig. 3). The best result was obtained using MEDBA: the concentration of mercury in the roots and stems was 44% and approximately 100%, respectively, higher than those of control plants, and the total mercury content in the plant increased by 2.8 times. A slightly smaller effect was observed from the combined treatment of sodium thiosulfate and PGRs (Ga/IAA/Fe-chelate). This complex led to a 20% increase in root mercury concentration, a 220% (2.2 times) increase in the shoots and a 210% (2.1 times) increase in the whole plants in comparison to those of the control.

Notably, as a result of the combination of MEDBA with phytohormones and iron chelate, as in the case of plant growth and development, the absorption and total accumulation of mercury decreased compared to those associated with the use of a chemical reagent alone. Mercury concentration decreased by 19.5% in shoots and 14.7% in roots; the accumulation of mercury in shoots decreased by 36% and in roots by 4%.

**Fig. 4:**
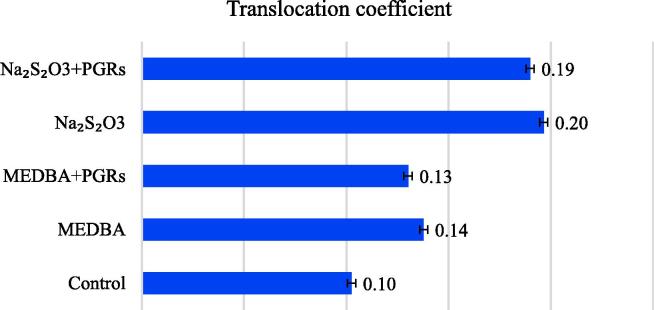
Translocation coefficients of mercury when using various induction complexes in phytoextraction.

When additives were combined, an inhibitory effect of the phytohormone complex relative to the action of MEDBA and a conspicuous increase in the action of thiosulfate was observed. Additionally, the chemical features of the chelating agent were reflected in the translocation coefficient values. The diagram (Fig. 4) shows that the highest efficiency of pollutant transfer to terrestrial plant parts was verified in plants treated with thiosulfate, providing a translocation coefficient of 0.19–0.20, twice higher than that of control plants. The schemes of mercury phytoextraction using MEDBA exhibited a small increase in translocation. However, the lower translocation coefficients with MEDBA compared with those of thiosulfate were not due to a lower accumulation of mercury in the watercress shoots but due to a higher accumulation of the pollutant in the roots.

In general, the experimental data support the following sequence of effectiveness in assisting mercury phytoextraction:

MEDBA > Na_2_S_2_O_3_ + PGRs > MEDBA + PGRs > Na_2_S_2_O_3_

## 4 Discussion

Studies have revealed a pronounced effect of chelating agents on the efficiency of mercury phytoextraction. MEDBA belongs to the class of complexone derivatives (synthetic polycarboxylic acids), and its presence in the compound determines the high biological activity of the reagent. As mentioned above, monoethanolamine exhibits growth-regulating and adaptogenic properties during plant development (Ilker et al., 1976, Eckert et al., 1988). In such cases, a good physiological state was observed in watercress sprouts as well as increased biomass at a given level of mercury contamination (5 MPC). The pre-determined optimal concentration of MEDBA (at a ratio of 1 ml of MEDBA to 100 ml of distilled water) on the culture of *T. repens* also proved effective for *L. sativum* watercress, suggesting that these established concentrations should be used with other plants when testing for mercury phytoextraction. Overall, MEDBA was more effective than the most common ligand. Thiosulfate, even when used in combination with exogenous phytohormones and iron chelate (GA/IAA/Fe-EDDHA). The results corroborate with previously published results, both in relation to the use of thiosulfates as corrections and to complexones (EDTA, DTPA). As reported, contrasting results were reported regarding mercury phytoextraction, ligand concentrations, and type of phytoextractor plants used. According to results of Moreno et al. (2004; 2005) from tests on native soil collected in the mine area Tui (New Zealand), using *Brassica juncea*, and adding (NH_4_)_2_S_2_O_3_, mercury absorption varied from 6.7- to 12.2-fold. More recent reports describe 53-fold [(NH_4_)_2_S_2_O_3_ versus 35.45-fold (Na_2_S_2_O_3_)] (Wang et al., 2017) and 28.4-fold (Grifoni et al., 2017) amplification. These results were obtained at significantly high values of mercury content in soil substrates (from 15.1 to 374 mg/kg soil DW) and high applied concentrations of the inductor. However, in experiments performed using sarepta mustard (*B. juncea*), oxalis corniculata (*Oxalis corniculata*), and vetiver (*Chrysopogon zizanioides*) (Lomonte et al., 2014, Liu et al., 2018a, Liu et al., 2018b), the increase in the absorption rate was not as high as that reported by Grifoni et al., (2017); the absorption rate was amplified by 1.06-, 1.63-, and 1.68– fold, respectively, using ammonium thiosulfate when compared to that of the control. Smolińska and Cedzyńska (2007) studied mercury absorption by watercress (*Lepidium sativum*) and reported a slight enhancement (as much as 1.04-fold), considering the concentration of mercury in the substrate from 2 to 20 mg/kg DW and the concentration of the added chelating agent (1 g/kg). In a previous study (Rodriguez et al., 2016), a 42% increase in mercury absorption by white lupine (*Lupinus albus*) was observed after adding a chelating agent at a concentration of 1 g/kg DW. Additionally, mercury phytoextraction using *O. corniculata* with two complexones, EDTA and DTPA, was confirmed in a screening comparison (Liu et al., 2018a, Liu et al., 2018b). The most significant enhancement effects for EDTA-induced mercury phytoextraction were observed by Nejatzadeh-Barandozi et al. (2014) testing amaranth (*Amaranthus retroflexus*), sorghum bicolor (*Sorghum bicolor*)*,* and perennial ryegrass (*Lolium perrene*), and they verified a 12.8-, 5.5- and 6- fold phytoextraction amplification, respectively. In all the three-plant species. EDTA treatment not only resulted in a significant increase in mercury absorption but also in a high level of translocation into the shoots.

Thus, MEDBA can be characterised as a highly effective reagent; however, more extensive testing is needed to understand various aspects of its action, including leaching and action on native contaminated substrates.

The combination of chemical amendments with a complex of exogenous growth regulators (GA/IAA/Fe-EDDHA) resulted in a synergistic effect in combination with thiosulfate but inhibited the action of MEDBA. As in a previous study (Cassina et al., 2012) in which assisting the phytoextraction of mercury from contaminated soils with thiosulfate was supplemented by treatment with cytokinins and other PGRs (GA/IAA/Fe-EDDHA), it also led to an increase in the effect of thiosulfate and achieved increases in mercury uptake by 2.48 and 2.32 times for Indian mustard (*Brassica juncea*) and sunflower (*Helianthus annuus*), respectively. For example, the total accumulation increased by additional 30% and 212%, respectively, compared with the control. The results of previous studies (Cassina L et al., 2012) as well as that of the present study showed a positive effect from a combination of various exogenous phytohormones and thiosulfate. Such schemes have great potential for practical applications. The increasing effect of the chelating agent by treatment with GA and IAA was also recorded in relation to other inducers: EDTA with phytoextraction of lead using corn (*Zea mays* L.) and HEDP for the phytoextraction of mercury (Hadi et al., 2010), cadmium, copper, and nickel (Makarova et al., 2021a, 2021b) with *T. repens.* These data corroborate with the results obtained using the induction scheme [thiosulfate + PGRs (GA/IAA/Fe-EDDHA)], although complexones of different classes were used (polyaminopolycarboxylic and phosphonic acids). Regarding MEDBA, a pronounced antagonistic physiological effect was observed between exogenous PGRs (GA/IAA/Fe-EDDHA) and monoethanolamine groups. Presently, it is still difficult to provide a detailed explanation of the mechanism of this phenomenon because the metabolic features of monoethanolamine and its role in the hormonal status of plants are unclear. Alternatively, MEDBA can likely be characterised as a double-acting chelating agent that increases the availability of mercury ions for absorption by plants as well as the physiological limit of their endurance.

## 5 Conclusions

This study demonstrated the high efficacy of the new chelating agent MEDBA and expanded our current understanding of its action. The optimal concentration ranges are average and low levels, at which the stimulating effect of monoethanolamine, a part of the molecular composition of the compound, is manifested. Concomitantly, antagonism between monoethanolamine and exogenous PGRs (GA/IAA/Fe-EDDHA) was observed when combined treatments resulted in slightly decreased plant growth, absorption, and mercury accumulation in plant parts. Unlike MEDBA, the use of a combined induction scheme with thiosulfate (thiosulfate + PGRs) was synergistic, increasing the effect of mercury extraction from contaminated soil substrates.

The results obtained validate MEDBA as a chelating agent for practical use in mercury phytoextraction, along with the already known corrections—thiosulfates (sodium or ammonium thiosulfate) and polyaminopolycarboxylic acids (EDTA and DTPA). However, the possibilities of this compound need to be further investigated to understand the limits of its action, for example, in extremely high concentrations of a pollutant in the environment. For example, in Russia, on the Southern Baikal region, the chemical enterprise Usoliekhimprom LLC operated until 2017, but it continues to be one of the main sources of serious mercury pollution. The contamination covers more than 600 ha, and the concentration of mercury in the soil substrate can reach 200 MPC. A similar situation is observed in the vicinity of the Wanshan mercury mine (Guangzhou, China).

The formula of the MEDBA compound from the class of dicarboxylic acids was proposed based on the presence of sulfur atom in the molecule, which ensures the high selectivity of the reagent to mercury. Achievements of modern chemistry of complex compounds make it possible not only to select a chelating agent of suitable properties from known chemical formulas, but also to purposefully obtain a given design of a molecular structure as the next step. In chelate-assisted phytoextraction of mercury, based on dithiocarboxylic acids, whose derivatives include MEDBA, it is possible to develop and synthesise a dual-action reagent containing heavy metal coordination zones of different nature in the molecular structure: iminodiacetate and thioacetate groups along with the hydroxyl group. Such a formula can provide high complexing activity to mercury and ions of other heavy metals and high solubility in water. Thus, under soil conditions, the chelating effect can simultaneously manifest itself selectively toward mercury and other accompanying heavy metals. In native soils contaminated with heavy metals, a pool of heavy metals was almost always present. The creation and use of a mixed ligand with the indicated properties would allow optimising the phytoextraction process to reduce the number of vegetation cycles for the extraction of various types of metals and to increase the integral coefficient of bioconcentration.

## Declaration of Competing Interest

The authors declare that they have no known competing financial interests or personal relationships that could have appeared to influence the work reported in this paper.
